# Retention and Fouling during Nanoparticle Filtration: Implications for Membrane Purification of Biotherapeutics

**DOI:** 10.3390/membranes12030299

**Published:** 2022-03-07

**Authors:** Liang-Kai Chu, S. Ranil Wickramasinghe, Xianghong Qian, Andrew L. Zydney

**Affiliations:** 1 Department of Chemical Engineering, The Pennsylvania State University, University Park, PA 16802, USA; lxc5534@psu.edu; 2 Ralph E. Martin Department of Chemical Engineering, University of Arkansas, Fayetteville, AK 72701, USA; swickram@uark.edu; 3 Department of Biomedical Engineering, University of Arkansas, Fayetteville, AK 72701, USA; xqian@uark.edu

**Keywords:** viral vectors, nanoparticles, adeno-associated virus, fouling, hydrophobicity

## Abstract

One major challenge in the development of nanoparticle-based therapeutics, including viral vectors for the delivery of gene therapies, is the development of cost-effective purification technologies. The objective of this study was to examine fouling and retention behaviors during the filtration of model nanoparticles through membranes of different pore sizes and the effect of solution conditions. Data were obtained with 30 nm fluorescently labeled polystyrene latex nanoparticles using both cellulosic and polyethersulfone membranes at a constant filtrate flux, and both pressure and nanoparticle transmission were evaluated as a function of cumulative filtrate volume. The addition of NaCl caused a delay in nanoparticle transmission and an increase in fouling. Nanoparticle transmission was also a function of particle hydrophobicity. These results provide important insights into the factors controlling transmission and fouling during nanoparticle filtration as well as a framework for the development of membrane processes for the purification of nanoparticle-based therapeutics.

## 1. Introduction

Recent advances in gene therapy have created renewed interest in the development of biological and synthetic nanoparticle systems for the delivery of RNA and DNA therapeutics. Liposomes and lipid nanoparticles protect nucleic acids from degradation and improve pharmacokinetics [1], forming the basis for mRNA vaccines against COVID-19. Recombinant viral vectors, including both lentivirus [2] and adeno-associated virus (AAV) [3], provide high transfection levels to effectively deliver mRNA and DNA to specific target organs [4]. In addition, nanoparticles can be used to treat solid tumors, e.g., through the generation of hydroxyl radicals that inhibit tumor growth [5].

One challenge in the development of nanoparticle-based therapeutics is the development of effective purification schemes. This includes ensuring sterility of the final product. Frequently, low yields are reported during filtration through sterilizing-grade membranes [6,7]. There is also a need to separate empty from full (DNA- and RNA-containing) capsids [8,9,10], which have similar physical characteristics. 

Density gradient centrifugation allows for the effective purification of viral capsids on a small scale, but this technique is difficult to apply for large-scale processing [11]. Size-exclusion (SEC) and ion-exchange (IEX) chromatography have both been explored for virus purification, but the large size of the nanoparticles leads to low binding capacities and significant mass transfer limitations [12,13]. 

Thus, there is much interest in the potential for using membrane technology specifically targeted at the purification of nanoparticle-based therapeutics. Arunkumar and Singh [14] examined the use of tangential flow ultrafiltration (TFF) for the final concentration and formulation (buffer exchange) of an AAV product and obtained good results using a 30 kDa nominal molecular weight cutoff composite regenerated cellulose membrane. Peixoto et al. [15] examined the potential of a fully membrane-based downstream process for adenovirus purification. Initial clarification was performed using normal flow filtration through a 0.8 µm prefilter and a 0.45 µm membrane with the clarified harvest concentrated by TFF. This was followed by an anion-exchange membrane adsorber and then final concentration and formulation by ultrafiltration (UF). The best performance for the TFF was obtained using a 300 kDa polysulfone membrane, as the larger molecular weight cutoff (500 and 750 kDa) membranes showed inadequate adenovirus retention. 

In addition to these studies of viral vector purification, a number of investigators have looked at the ultrafiltration of model nanoparticles that may be significant environmental toxins. For example, Le Hir et al. [16] evaluated the effects of salinity and polydispersity on the ultrafiltration of small (1.5 and 10 nm) nanoparticles and noted significant effects of intermolecular interactions between the nanoparticles and with the membrane. Jassby et al. [17] examined the filtration of C60 fullerenes in the presence of different salts, showing that the addition of divalent cations led to the formation of large aggregates that were more highly retained by a ceramic membrane.

Despite these previous efforts, there is still little fundamental information on the factors controlling nanoparticle filtration. The objective of this study was to evaluate the retention and fouling behavior of 30 nm polystyrene nanoparticle suspensions as a model for an AAV biotherapeutic. Data were obtained over a range of solution conductivities and surfactant concentrations for nanoparticles with different surface charge and hydrophobicity using both cellulosic and polyethersulfone membranes, the two most widely used polymeric membranes in bioprocessing [18]. Our results provide important insights into the factors controlling nanoparticle filtration, providing a framework for future studies of membrane systems for the purification of nanoparticle-based biotherapeutics.

## 2. Materials and Methods

### 2.1. Nanoparticle Feed Solution Preparation

Data were obtained with 30 nm fluorescently labeled polystyrene latex nanoparticles from MagSphere (Pasadena, CA, USA) as a model for AAV (reported size of 25–28 nm [19,20]). Three different nanoparticles were examined with different dyes (blue, red, and orange); these nanoparticles had similar size but different hydrophobicity, as discussed in the Results. Nanoparticles were suspended at a concentration of approximately 3.4 × 10^12^ particles/mL in 10 mM Tris buffer prepared from a 1 M Tris stock solution (Invitrogen, Waltham, MA, USA) using deionized (DI) water from a Millipore Direct-Q purification unit (Burlington, MA, USA). Solution pH was adjusted to 7.5 using 1 M HCl. Tween 20 (0.01 weight percent) (Sigma, St. Louis, MO, USA) was added to minimize particle aggregation. Conductivity was adjusted by addition of NaCl (RND Center, La Jolla, CA, USA). 

The particle size distribution and zeta potential were evaluated using a Zetasizer Nano ZS90 (Malvern Panalytical, Westborough, MA, USA). Samples were prepared in 10 mM Tris buffer with 0.01% Tween 20 without sonication. The light-scattering intensity was measured for three repeat scans at room temperature. The particle size was determined from the measured diffusivity based on the Stokes–Einstein equation, while the zeta potential was determined from the measured electrophoretic mobility.

### 2.2. Modified Hydrophobic Interaction Chromatography

Nanoparticle hydrophobicity was evaluated using the membrane hydrophobic interaction chromatography (MHIC) protocol developed by Taylor et al. [21]. Nanoparticle retention was determined using a 5 μm pore size polyvinylidene fluoride (PVDF) Durapore membrane from MilliporeSigma (Burlington, MA, USA) as the solid phase. The membrane was initially equilibrated in 2 M ammonium sulfate, 50 mM phosphate buffer at a flow rate of 4 mL/min (corresponding to a linear velocity of 0.32 mm/s) set using an AKTA 150 fast protein liquid chromatography (FPLC) system (Cytiva, Marlborough, MA, USA). A 1 mL nanoparticle suspension was injected, and nanoparticle elution was evaluated using a linear gradient between the equilibration buffer and a 5 mM phosphate buffer (from 0% to 100% phosphate buffer) over 15 min. The nanoparticle concentration was determined by UV absorbance at 280 nm.

### 2.3. Nanoparticle Filtration

Filtration experiments were performed with 0.1 μm pore size mixed cellulose ester microfiltration membranes; 100 and 300 kDa nominal molecular weight cutoff Ultracel composite regenerated cellulose ultrafiltration membranes; and 100, 300, and 500 kDa Biomax polyethersulfone membranes, all provided by MilliporeSigma (Burlington, MA, USA). Cellulosic and polyethersulfone membranes are widely used in bioprocessing for the purification of recombinant proteins, monoclonal antibodies, and vaccines [18]. Cellulosic membranes are highly hydrophilic (with contact angle < 20°), while polyethersulfone membranes are somewhat more hydrophobic (contact angle of 56° [22]). The mixed cellulose ester membrane was simply flushed with DI water to remove any storage or wetting agents, while the Ultracel and Biomax membranes were first soaked in isopropyl alcohol (IPA) before flushing. Membranes were cut into 25 mm diameter discs and placed in the base of a stainless steel holder (Pall, New York, NY, USA). 

The nanoparticle suspension was fed to the filtration unit using a Masterflex peristaltic pump (Cole-Parmer, Vernon Hill, IL, USA) operated at a filtrate flow rate of 0.93 mL/min, providing a filtrate flux of 150 L/m^2^/h (LMH). PTFE tubing was used for all connections to minimize particle adhesion and loss. Transmembrane pressure was evaluated using a digital pressure gauge (Ashcroft, Stratford, CT, USA) placed immediately upstream of the stainless steel holder. 

Permeate samples were obtained every five minutes over the first twenty minutes and then every ten minutes until the end of the filtration. Nanoparticle concentrations were measured in duplicate based on the fluorescence intensity evaluated using 96-well Fisher black-bottom plates placed in a Tecan Infinite 200 microplate reader (Hombrechtikon, Switzerland). The fluorescence intensity was highly linear over the full range of particle concentrations with R^2^ > 0.99. 

## 3. Results and Discussion

### 3.1. Nanoparticle Characterization

**Figure 1:**
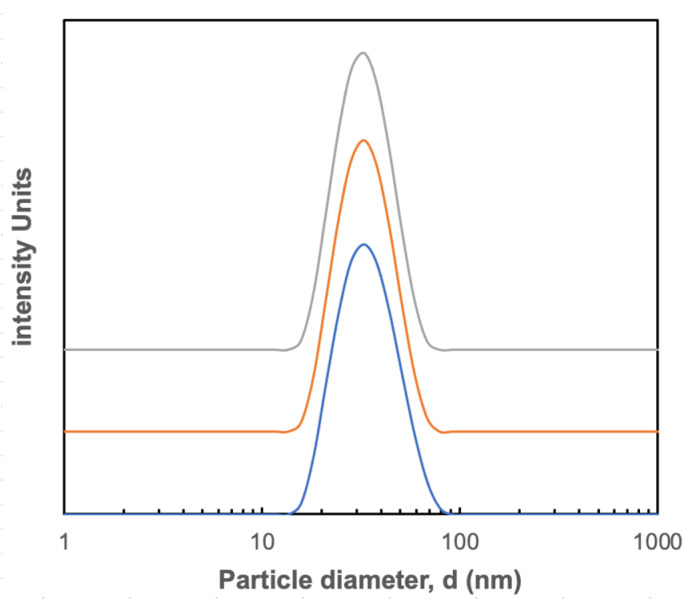
Intensity distribution of blue polystyrene nanoparticles determined using dynamic light scattering. Different color curves are shown for 3 repeat measurements with the y-axis displaced to improve clarity.

Figure 1 shows the size distribution of the suspension of blue nanoparticles obtained by dynamic light scattering (DLS). The suspension was monodisperse with a Z-average size of 34 nm and a range from 15 to approximately 80 nm. Similar results were obtained with the orange and red nanoparticles (Z-average size provided in Table 1). The measured size was slightly larger than that provided by the manufacturer (28–30 nm) but was consistent with limited SEM images (Figure S1). There was no evidence of any nanoparticle aggregation. The measured size was similar to the range reported for AAV [19,20]. The nanoparticles were all negatively charged; the measured zeta potentials were around −15 mV for the blue, orange, and red nanoparticles, as summarized in Table 1. 

**Figure 2:**
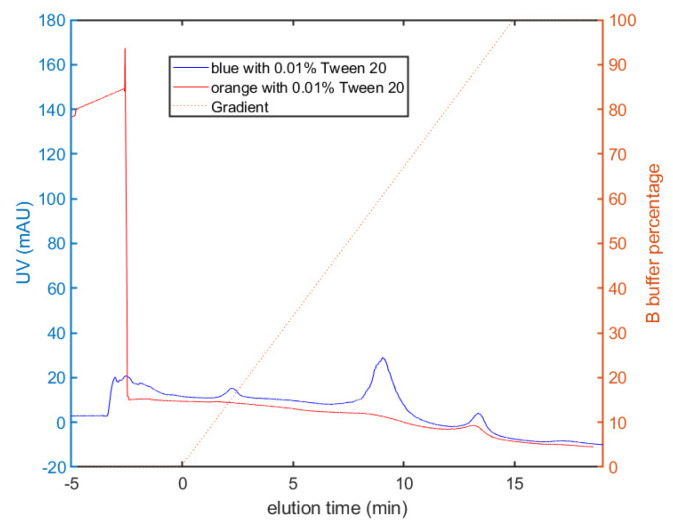
Membrane hydrophobic interaction chromatography for orange and blue nanoparticles.

The surface hydrophobicity of the polystyrene nanoparticles was determined by membrane hydrophobic interaction chromatography using a 5 µm pore size PVDF membrane as the solid phase. Typical results are shown in Figure 2 for the blue and orange nanoparticles in response to a linear gradient between 2 M ammonium sulfate with 50 mM phosphate and 2 M ammonium sulfate with 5 mM phosphate, both with 0.01% Tween 20. The blue particles showed three distinct peaks: a larger peak at 8 min (corresponding to approximately 60% of the 5 mM phosphate buffer) and smaller peaks at 2 and 13 min. In contrast, the orange nanoparticles showed only a single peak at 13 min near the end of the gradient, i.e., at a greater percentage of phosphate for elution. This indicated that the orange nanoparticles were significantly more hydrophobic than the blue nanoparticles. These differences in hydrophobicity were likely related to the properties of the fluorescent dyes used to label the different nanoparticles.

### 3.2. Nanoparticle Filtration

**Figure 3:**
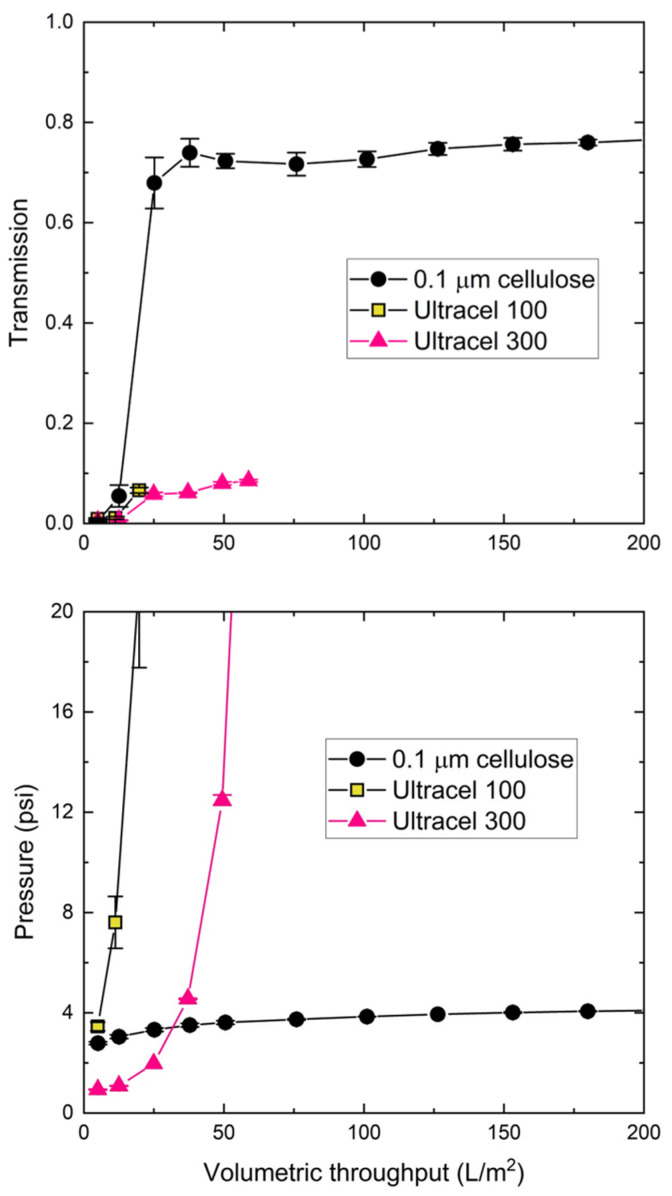
Particle transmission (**top panel**) and transmembrane pressure (**bottom panel**) during filtration of 30 nm blue polystyrene nanoparticles through the different cellulosic membranes at a concentration of 3.4 × 10^12^ particles/mL in 10 mM Tris buffer with 0.01% Tween 20 at a constant filtrate flux of 150 LMH. Error bars represent the standard deviation for repeat measurements (not shown when smaller than the size of the symbol).

The cellulosic membranes of different pore sizes were challenged with the 30 nm blue polystyrene nanoparticles at a constant flux of 150 LMH, with the data for transmembrane pressure (TMP) and nanoparticle transmission as a function of the volumetric throughput shown in the bottom and top panels of Figure 3, respectively. The data were highly reproducible, as repeat experiments showed nearly identical behavior with <10% differences in both the TMP and transmission. Both the Ultracel 100 and 300 membranes showed rapid fouling, with pressure exceeding 20 psi (140 kPa) after approximately 25 and 50 L/m^2^, respectively. The nanoparticle transmission, defined as the ratio of the nanoparticle concentration in the permeate samples to that in the feed, was evaluated as:

(1) $$T=\frac{I_{permeate}}{I_{feed}}$$

where *I_permeate_* and *I_feed_* are the fluorescence intensities in the permeate and feed samples, respectively. The nanoparticle transmission for the Ultracel 100 and 300 membranes remained below 10% over the course of the filtration, which was consistent with the small effective pore size of these membranes (see Table 2). In contrast, the 0.1 μm mixed cellulose ester membrane had >70% particle transmission, and the transmembrane pressure stabilized at approximately 4 psi. The increase in nanoparticle concentration in the permeate samples over the first 20 L/m^2^ was likely due, in part, to dilution effects associated with the hold-up volume within the membrane and module, although there may also have been a low level of nanoparticle retention during the initial stage of the filtration. There was no evidence of any selective retention of the nanoparticles by the 0.1 µm mixed cellulose ester membrane; the mean size of the nanoparticles in the permeate samples was statistically identical to that for the feed based on DLS.

Table 2 summarizes the results for the Ultracel, Biomax, and mixed cellulose ester membranes challenged with the blue nanoparticle suspensions in 10 mM Tris buffer at a pH of 7.5. The pore diameters (*d_p_*) for the ultrafiltration membranes were calculated from the measured hydraulic permeabilities (*L_p_)* of the membranes as [23]:

(2) $$d_{p}={\left(\frac{32µ\delta_{m}L_{p}}{\varepsilon }\right)}^{1/2}$$

where µ is the solution viscosity, $\delta_{m}$ is the thickness of the membrane skin (taken as 1 µm), and *ε* is the membrane porosity. Equation (2) may underestimate pore size, particularly for the Biomax 500 kDa membrane, as it does not account for the resistance of the membrane substructure. The 100 and 300 kDa nominal molecular weight cutoff membranes fouled rapidly with very low nanoparticle transmission, which was consistent with the small pore size of these membranes. The results for the Ultracel (cellulose) and Biomax (polyethersulfone) membranes were similar, despite differences in polymer chemistry. The 0.1 μm mixed cellulose ester and Biomax 500 membranes had relatively little fouling with more than 70% nanoparticle transmission (evaluated over the entire filtration experiment). In addition, the transmembrane pressure remained below 5 psi up to 200 L/m^2^.

### 3.3. Buffer Effects

**Figure 4:**
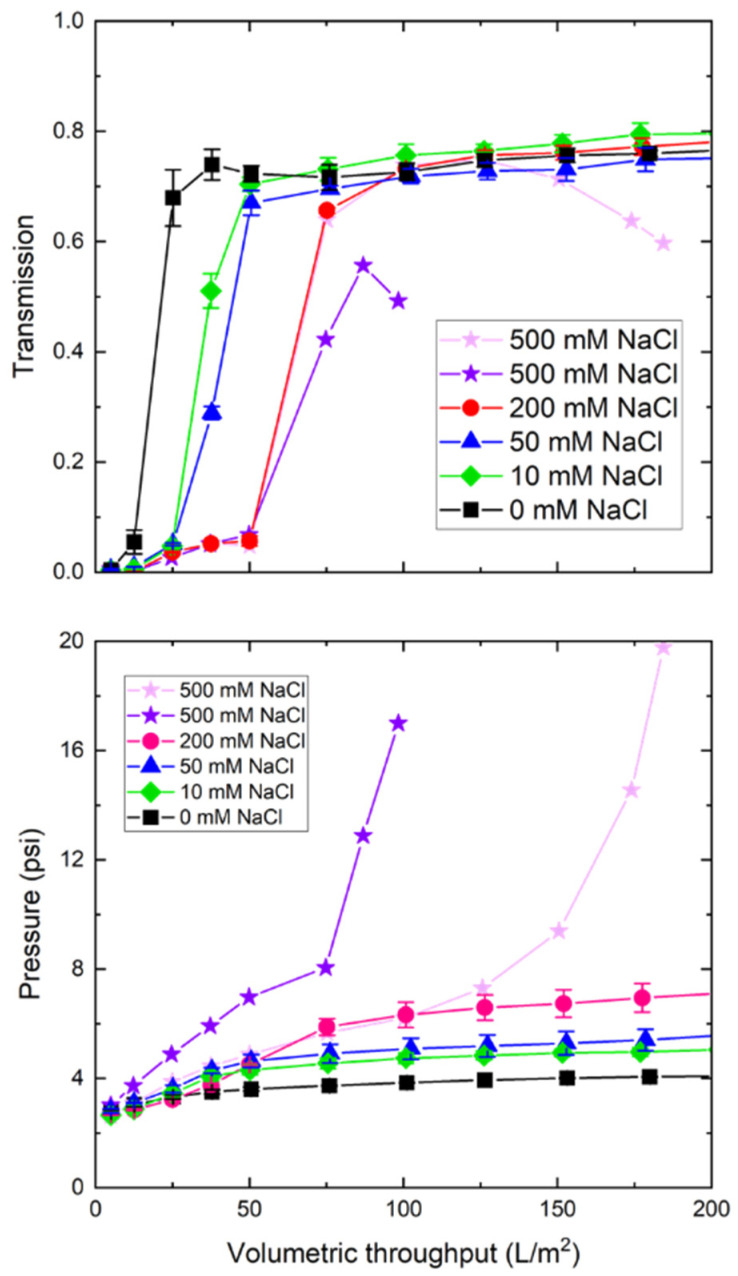
Nanoparticle transmission (**top panel**) and transmembrane pressure (**bottom panel**) during filtration of the 30 nm blue polystyrene nanoparticles through 0.1 μm mixed cellulose ester membranes in the presence of added NaCl at a constant filtrate flux of 150 LMH.

Figure 4 examines the effects of buffer conductivity, adjusted by the addition of NaCl, on the TMP and nanoparticle transmission through the 0.1 μm mixed cellulose ester membrane. The transmission increased rapidly for the solution without any added NaCl, attaining a value above 70% after only 30 L/m^2^. Similar profiles were obtained in the presence of 10, 50, and 200 mM NaCl, while the data for the higher NaCl solutions showed a delay in the rise in transmission. This effect was quite pronounced for the 200 mM NaCl solution, as the transmission remained below 6% over the first 50 L/m^2^ and then rapidly increased to approximately 70%. This time lag was likely due to nanoparticles binding to the filter in the higher ionic strength solutions, which was consistent with the greater increase in transmembrane pressure (bottom panel). The steady-state transmission at high throughput may reflect the saturation of the binding sites within the membrane. The behavior in the presence of 500 mM NaCl was quite different. In both repeat experiments, the nanoparticle transmission passed through a maximum before declining at high throughput. In addition, the TMP showed an intermediate plateau followed by a rapid increase to more than 15 psi. This may have reflected the formation of a particle cake on or within the 0.1 µm mixed cellulose ester membrane, which served as a dynamic membrane that was able to retain the nanoparticles in the feed.

**Figure 5:**
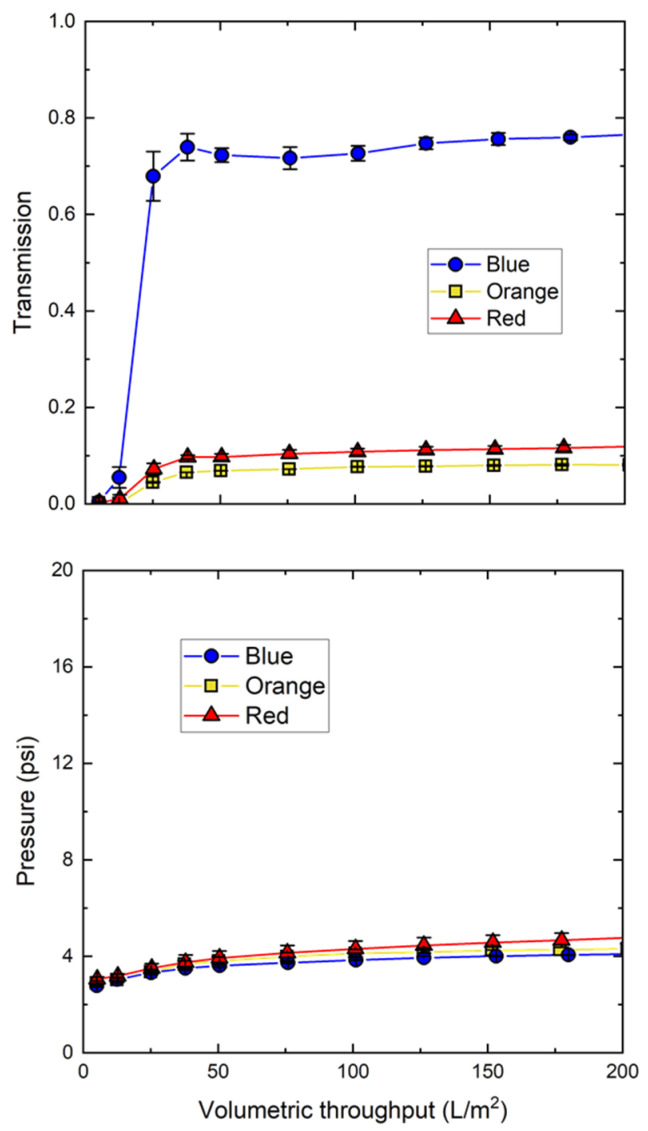
Particle transmission (**top panel**) and transmembrane pressure (**bottom panel**) for the different 30 nm polystyrene nanoparticles during filtration through 0.1 µm mixed cellulose ester membranes at a constant filtrate flux of 150 LMH. Data obtained with suspensions containing 3.4 × 10^12^ particles/mL in 10 mM Tris buffer with 0.01% Tween 20.

The effect of nanoparticle hydrophobicity on filtration behavior is examined in Figure 5, which shows data for the filtration of the blue, orange, and red nanoparticles through separate 0.1 µm mixed cellulose ester membranes. In contrast to the blue nanoparticles, which showed nearly 80% transmission, the orange and red nanoparticles showed a maximum transmission of only 10%, even though the TMP remained below 5 psi up until 200 L/m^2^. This is very different than the behavior observed with the 100 and 300 kDa Ultracel membranes, which retained the nanoparticles due to their small pore size. Instead, the low transmission of the orange and red nanoparticles by the 0.1 µm pore size membrane was likely due to hydrophobic interactions, which is consistent with the greater hydrophobicity (retention time) evaluated in the MHIC analysis (Table 1). 

To confirm that the behavior seen in Figure 5 was not due to differences in the membranes used in the different experiments, data were obtained with a binary mixture of the blue and orange nanoparticles using the Biomax 500 kDa membrane (Figure S2). The nanoparticle transmission data for the binary mixture were nearly identical to those obtained with suspensions of the individual nanoparticles, as there was high transmission of the more hydrophilic blue nanoparticles and high retention of the more hydrophobic orange nanoparticles. The transmembrane pressure increased somewhat more rapidly during filtration of the binary mixture, which may reflect some degree of particle–particle interactions with the more hydrophobic polyethersulfone membrane, or it may simply reflect the inherent run-to-run variability.

**Figure 6:**
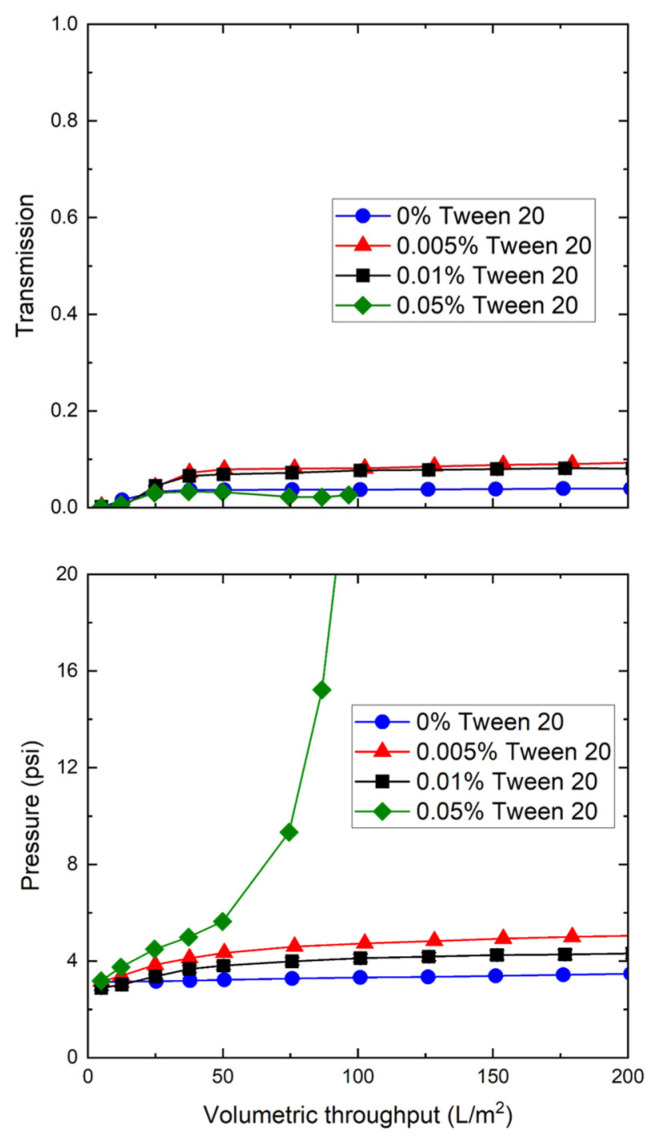
Effect of Tween 20 on nanoparticle transmission (**top panel**) and transmembrane pressure (**bottom panel**) during filtration of the 30 nm orange polystyrene nanoparticles through 0.1 µm mixed cellulose ester membranes at a constant filtrate flux of 150 LMH. Data obtained with suspensions having 3.4 × 10^12^ particles/mL in 10 mM Tris buffer.

Additional insights into the effects of hydrophobic interactions on nanoparticle transmission were obtained by performing a series of experiments with the very hydrophobic orange particles in the presence of different amounts of Tween 20. The addition of small amounts of Tween 20 caused a small increase in nanoparticle transmission, from <4% to approximately 8% as the Tween 20 concentration increased from 0% to 0.01% (Figure 6). However, the nanoparticle transmission in the presence of 0.05% Tween 20 remained below 4% throughout the experiment, and the TMP increased rapidly, providing a maximum capacity of <100 L/m^2^. This was unlikely to be due to micelle formation as the critical micelle concentration for Tween 20 is reported as being between 0.06% and 0.07% [24]. In addition, this rapid increase in TMP was not seen when 0.05% Tween 20 was added to the less hydrophobic blue nanoparticles (Figure S3). Instead, the addition of Tween 20 may have been facilitating the aggregation and association of the more hydrophobic nanoparticles. Future studies are required to quantify this phenomenon.

## 4. Conclusions

This paper examined the effects of solution conditions and nanoparticle hydrophobicity on the filtration of 30 nm polystyrene nanoparticles through both cellulosic (highly hydrophilic) and polyethersulfone membranes. High nanoparticle transmission was obtained with both a 0.1 µm mixed cellulose ester membrane and a 500 kDa Biomax polyethersulfone membrane; both the 100 and 300 kDa Ultracel and Biomax membranes had high particle retention due to their small pore size, despite their difference in hydrophobicity. The addition of NaCl caused a delay in nanoparticle transmission through the 0.1 µm mixed cellulose ester membrane, while the use of 500 mM NaCl led to a decrease in transmission at high throughput. The filtration of more hydrophobic nanoparticles led to significantly greater fouling with relatively low nanoparticle transmission. The filtration of actual AAV particles is far more complex due to the variability of biological particles, the presence of a diverse array of impurities, and the need to separate filled from empty and defective capsids. The results obtained in this study provide important insights into the factors controlling nanoparticle retention and fouling, providing an initial framework that may be used to assist the development of membrane filtration systems for the purification of nanoparticle-based therapeutics like AAV. 

## Figures and Tables

**Table 1:** Physical properties of different nanoparticles used in this study.

	Emission Wavelength	Z-Average Diameter	Zeta Potential	MHIC Retention
Blue	419 nm	34 ± 2 nm	−15 ± 1 mV	2/8/13 min
Orange	614 nm	35 ± 1 nm	−15 ± 1 mV	13 min
Red	630 nm	35 ± 1 nm	−14 ± 1 mV	ND

**Table 2:** Pore size, throughput (evaluated at TMP = 10 psi), and average transmission for 0.1 µm mixed cellulose ester, Ultracel, and Biomax membranes.

	0.1 μm	Biomax 100	Biomax 300	Biomax 500	Ultracel 100	Ultracel 300
Pore diameter *d_p_* (nm)	100	13 ± 1	18 ± 1	32 ± 2	9.0 ± 0.2	20 ± 1
Throughput at 10 psi (L/m^2^)	>200	23	24	>200	13	46
Average Transmission	74%	5%	13%	79%	3%	5%

## Data Availability

The data presented in this study are available upon request from the corresponding author.

## Supplementary Materials

The following supporting information can be downloaded at: https://www.mdpi.com/article/10.3390/membranes12030299/s1, Figure S1: SEM image of polystyrene nanoparticles; Figure S2: Filtration of blue and orange nanoparticles, alone and in binary mixture, through the Biomax 500 kDa membrane; Figure S3: Effect of Tween 20 on the filtration of 30 nm blue nanoparticles through the 0.1 µm mixed cellulose ester membranes.
