# Supplementary material for: Retention and Fouling during Nanoparticle Filtration: Implications for Membrane Purification of Biotherapeutics
Source: Membranes (Basel). 2022 Mar 7;12(3):299. doi: 10.3390/membranes12030299 (PMC8953984; doi:10.3390/membranes12030299)
Supplement: Supplementary file 1 [file membranes-12-00299-s001.zip › membranes-1615993-supplementary.pdf]

membranes

Supplementary Materials

Retention and Fouling during Nanoparticle Filtration: Implications for Membrane Purification of Biotherapeutics

Liang-Kai Chu [1], S. Ranil Wickramasinghe [2], Xianghong Qian [3] and Andrew L. Zydney [1,*]

[1] Department of Chemical Engineering, The Pennsylvania State University, University Park, PA 16802, USA; lxc5534@psu.edu (L.-K.C.)
[2] Ralph E. Martin Department of Chemical Engineering, University of Arkansas, Fayetteville, AK 72701, USA; swickram@uark.edu (S.R.W.)
[3] Department of Biomedical Engineering, University of Arkansas, Fayetteville, AK 72701, USA;xqian@uark.edu (X.Q.)
* Correspondence: zydney@engr.psu.edu

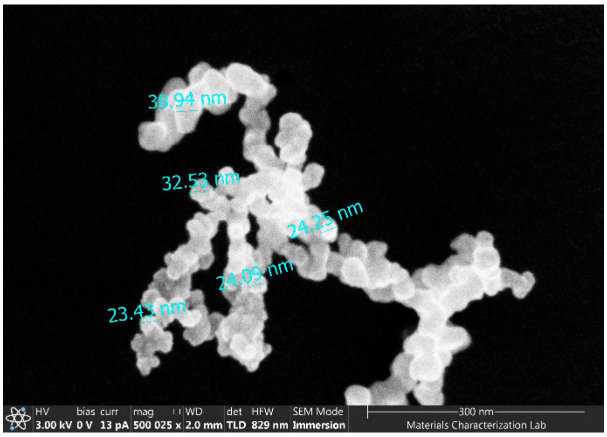

Figure S1. Image of polystyrene latex particles obtained by SEM (Thermo-Fisher Scientific APREO-S, U.S.A.). Particle suspension was air dried overnight on a silicon wafer and sputter-coated with iridium to generate an approximately 3 nm coating using an Emitech sputter coater (Quorum Technologies Ltd, UK). The particle aggregate is formed during the drying process.

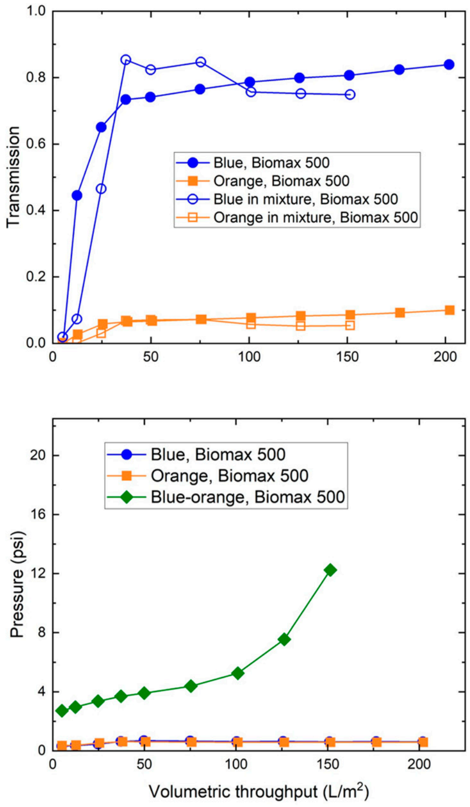

Figure S2. Particle transmission (top panel) and transmembrane pressure (bottom panel) for filtration of the blue and orange nanoparticles, both alone and in a binary mixture, through the 500 kDa Biomax membranes at a constant filtrate flux of 150 LMH. The binary mixture experiment was performed with 50% or each nanoparticle at the same total nanoparticle concentration as in the single nanoparticle experiments.

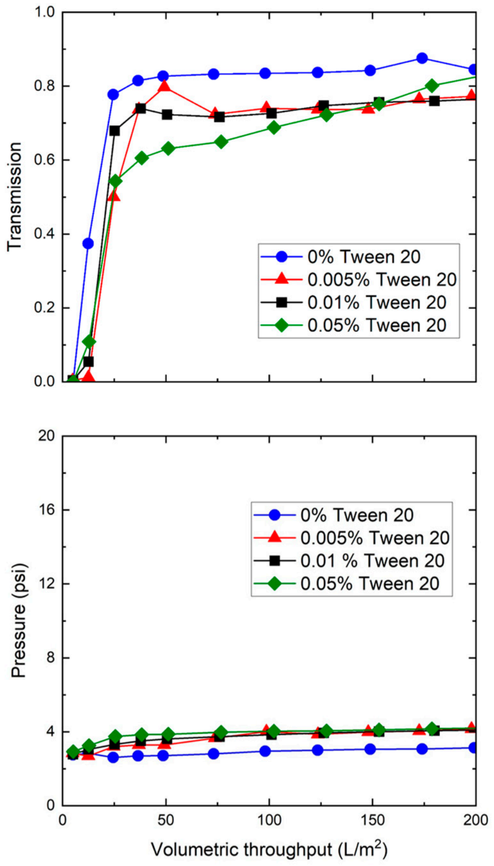

Figure S3. Effect of Tween 20 on nanoparticle transmission (top panel) and transmembrane pressure (bottom panel) during filtration of the 30 nm blue polystyrene nanoparticles through the 0.1 μm mixed cellulose ester membranes at a constant filtrate flux of 150 LMH. Data obtained with suspensions having 3.4×10^{12} particles/mL in 10 mM Tris buffer.
